# Risk prediction models for dementia: role of age and cardiometabolic risk factors

**DOI:** 10.1186/s12916-020-01578-x

**Published:** 2020-05-19

**Authors:** Aurore Fayosse, Dinh-Phong Nguyen, Aline Dugravot, Julien Dumurgier, Adam G. Tabak, Mika Kivimäki, Séverine Sabia, Archana Singh-Manoux

**Affiliations:** 1 Inserm U1153, Epidemiology of Ageing and Neurodegenerative diseases, Université de Paris, 10 avenue de Verdun, 75010 Paris, France; 2 First Department of Medicine, Semmelweis University Faculty of Medicine, Budapest, Hungary; 3 Department of Epidemiology and Public Health, University College London, London, UK

**Keywords:** CAIDE, Dementia risk score, Dementia, Cardiometabolic risk factors

## Abstract

**Background:**

Cardiovascular Risk Factors, Aging, and Incidence of Dementia (CAIDE) risk score is the only currently available midlife risk score for dementia. We compared CAIDE to Framingham cardiovascular Risk Score (FRS) and FINDRISC diabetes score as predictors of dementia and assessed the role of age in their associations with dementia. We then examined whether these risk scores were associated with dementia in those free of cardiometabolic disease over the follow-up.

**Methods:**

A total of 7553 participants, 39–63 years in 1991–1993, were followed for cardiometabolic disease (diabetes, coronary heart disease, stroke) and dementia (*N* = 318) for a mean 23.5 years. Cox regression was used to model associations of age at baseline, CAIDE, FRS, and FINDRISC risk scores with incident dementia. Predictive performance was assessed using Royston’s *R*^2^, Harrell’s C-index, Akaike’s information criterion (AIC), the Greenwood-Nam-D’Agostino (GND) test, and calibration-in-the-large. Age effect was also assessed by stratifying analyses by age group. Finally, in multistate models, we examined whether cardiometabolic risk scores were associated with incidence of dementia in persons who remained free of cardiometabolic disease over the follow-up.

**Results:**

Among the risk scores, the predictive performance of CAIDE (C-statistic = 0.714; 95% CI 0.690–0.739) and FRS (C-statistic = 0.719; 95% CI 0.693–0.745) scores was better than FINDRISC (C-statistic = 0.630; 95% CI 0.602–0.659); *p* < 0.001), AIC difference > 3; *R*^2^ 32.5%, 32.0%, and 12.5%, respectively. When the effect of age in these risk scores was removed by drawing data on risk scores at age 55, 60, and 65 years, the association with dementia in all age groups remained for FRS and FINDRISC, but not for CAIDE. Only FRS at age 55 was associated with dementia in persons who remained free of cardiometabolic diseases prior to dementia diagnosis while no such association was observed at older ages for any risk score.

**Conclusions:**

Our analyses of CAIDE, FRS, and FINDRISC show the FRS in midlife to predict dementia as well as the CAIDE risk score, its predictive value being also evident among individuals who did not develop cardiometabolic events. The importance of age in the predictive performance of all three risk scores highlights the need for the development of multivariable risk scores in midlife for primary prevention of dementia.

## Background

There is considerable evidence of the importance of vascular pathways to cognitive impairment and dementia [1–3]. The brain’s need for a constant supply of oxygen and glucose for maintenance of physiological function makes it vulnerable to vascular dysfunction [4]. Current understanding of Alzheimer’s disease, the primary cause of dementia, suggests that changes in biomarkers are present 15–20 years before the appearance of clinical symptoms [5]. Accordingly, there is emerging research on the association between midlife cardiometabolic risk factors and dementia although a wide age range, 35 to 68 years [6–9], is used to characterize midlife. Studies that have defined midlife with more precision have examined individual risk factors [10, 11], rather than the overall risk burden.

There are several multivariable risk scores for cardiovascular disease and diabetes that have been elaborated but the Cardiovascular Risk Factors, Aging, and Incidence of Dementia (CAIDE) risk score [12] is the only currently available midlife risk score specifically developed for dementia prediction. It is composed of sociodemographic and vascular risk factors: age, education, sex, systolic blood pressure, body mass index (BMI), total cholesterol, and physical activity. Age is the strongest known risk factor for dementia, and a previous paper found additional adjustment for age to attenuate the association between the Framingham cardiovascular Risk Score (FRS) [13], which already contains an age component, and dementia [14]. Furthermore, previous studies have not considered the fact that the association of cardiovascular risk factors with dementia depends on the age at assessment of cardiovascular risk burden, and mid- rather than late-life exposure has been shown to be important [10, 11, 15].

Our objective was to compare the association of CAIDE, FRS, and the FINDRISC diabetes [16] risk score with incidence of dementia. To address the effects of age in the risk scores, we examined the association of risk scores drawn from baseline (when participants were 39 to 63 years), adjusted for age, and then at ages 55, 60, and 65 years. This allowed us to test the hypothesis that when a wide age range is used at risk score assessment, the age component of the risk score is the prime driver of reported findings. We then examined whether the association of risk scores with dementia was mediated by clinical cardiovascular disease and diabetes.

## Methods

Data are drawn from the Whitehall II study, an ongoing prospective cohort study established in 1985 on 6895 men and 3413 women, aged 35 to 55 years at recruitment [17]. The study design consists of a self-administered questionnaire and a clinical examination every 4 to 5 years (1991–1993, 1997–1999, 2003–2004, 2007–2009, 2012–2013, and 2015–2016) which includes anthropometry, cardiovascular and metabolic risk factors, biochemical measures, and chronic diseases. Risk factors included in the analysis were incorporated in the study starting in 1991–1993; data for the construction of risk scores for each participant were drawn from 1991 to 1993 and from the closest wave of clinical examination when participants were 55, 60, and 65 years over the follow-up.

### Risk scores

CAIDE [12], FRS [13], and FINDRISC [18] risk scores were calculated using the original scoring methods (Additional file 1: Tables S1, S2, S3) at study baseline (age 39–63 years) and ages 55, 60, and 65 years for each participant. Venous blood was taken in the morning after ≥ 8 h of fasting or at least 5 h after a light, fat-free breakfast. Serum for lipid analyses was refrigerated at 4 °C and assayed within 72 h. Total cholesterol was measured using a Cobas Fara centrifugal analyzer (Roche Diagnostics System), HDL cholesterol by precipitating non-HDL cholesterol with dextran sulfate-magnesium chloride with the use of a centrifuge and measuring cholesterol in the supernatant fluid. Systolic blood pressure (mmHg) was taken as the average of two measurements (Hawksley random-zero sphygmomanometer) with the participant in a sitting position after 5 min rest. Treated hypertension was determined using chapters 2.2 to 2.6 of the British National Formulary. Diabetes was defined by a fasting glucose ≥ 7.0 mmol/L or reported doctor-diagnosed diabetes or the use of diabetes medication. Weight was measured in underwear to the nearest 0.1 kg on digital Soehnle electronic scales (Leifheit AS, Nassau, Germany). With the participant standing erect in bare feet with head in the Frankfurt plane, height was measured to the nearest 1 mm using a stadiometer. BMI (kg/m^2^) was calculated by dividing weight (in kilograms) by height (in meters squared). Waist circumference, the smallest circumference at or below the costal margin, was measured with subjects in the standing position in light clothing, using a fiberglass tape measure at 600 g tension.

Data on smoking status (current or never/ex-smoker), frequency of fruit and vegetables (8-point scale categorized as “less than daily” or “daily”), and education (years in full-time education) were reported by participants. Family history of diabetes was reported by the participants and personal history of diabetes ascertained from their clinical records in the study. Physical activity for the CAIDE was measured as engagement in activity causing sweating and breathlessness, at least twice a week, for a total weekly duration of 1 h or more. For the FINDRISC, it was duration in moderate to vigorous physical activity, at least 4 h a week [19].

### Incidence of coronary heart disease (CHD), stroke, and diabetes (1991–1993 to 2017)

CHD included non-fatal myocardial infarction, definite angina, coronary artery bypass grafting, and percutaneous transluminal coronary angioplasty. CHD ascertainment using data from in- or out-patient hospital consultations recorded in the national hospital episode statistics (HES) based on ICD-9 codes 410–414, ICD-10 codes I20–I25, or procedures K40–K49, K50, K75, U19.

Stroke cases were defined using ICD-9 codes 430, 431, 434, 436 and ICD-10 codes I60–I64 from HES records and self-reported stroke which was validated against medical records [20].

Diabetes diagnosis was based on fasting glucose ≥ 7.0 mmol/L (126 mg/dL), reported physician-diagnosed diabetes, use of diabetes medication, or HES record (ICD-9 codes 250 and ICD-10 code E11).

### Dementia ascertainment

All residents in the UK have a unique National Health Service (NHS) identification number which was used to link all participants to electronic health records. Three registers (HES, the Mental Health Services Data Set, and the mortality register) were used for dementia ascertainment using ICD-10 codes F00–F03, F05.1, G30, and G31. Record linkage was available until 31 March 2017. The NHS provides most of the health care, including out- and in-patient care. The sensitivity and specificity of dementia in the NHS HES data is 78.0% and 92.0% [21]. In addition, we used the Mental Health Services Data Set, a national database which contains information on dementia for persons in contact with mental health services in hospitals, out-patient clinics, and the community.

### Statistical analyses

The TRIPOD checklist is included in Additional file 1: Table S4.

### Risk scores and incidence of dementia

As there was no suggestion of deviations from linearity (Additional file 1: Fig. S1), all three risk scores were standardized (Mean = 0, SD = 1) to allow comparison between them, sex-specific for the FRS and FINSRISC in accordance with the original scoring. We used Cox proportional hazard regression to examine the predictive performance of the three risk scores, drawn from baseline assessment in 1991–1993. Participants were followed to the date of record of dementia, death, or 31 March 2017, whichever came first. Censoring participants who died over the follow-up at the date of death allowed us to account for competing risk of death using cause-specific hazard models [22].

Assumptions of proportional hazards and log-linearity were found not to be violated using Schoenfeld and Martingale residuals. For each risk score, we estimated Royston’s modified *R*^2^ for survival data as a measure of overall performance (higher values indicate a greater proportion of variation explained) along with confidence intervals calculated using 2000 bootstrap replications [23]; Harrell’s C-statistic for discrimination, [24] which were formally compared using a nonparametric approach [25]; the Akaike information criterion (AIC) for relative goodness-of where lower absolute values indicate better model fit, differences in AIC of 3 or more are considered to be meaningful; calibration for agreement between observed and predicted risk was tested using the Greenwood-Nam-D’Agostino (GND) test, an extension of the Hosmer-Lemeshow test, *p* < 0.05 indicates a lack-of-fit [26]; and calibration-in-the-large shown in plots of observed and predicted dementia rate per 1000 person/years in deciles of the predictor.

Subsequent analyses were stratified by age at exposure (ages 55, 60, and 65 years) and the predictive performance of CAIDE was compared to the FRS and FINDRISC. The follow-up time in these analyses was calculated from age at exposure (ages 55, 60, and 65 years) to the record of dementia, death, or March 31, 2017, whichever came first. In a complimentary approach to assessing the role of age, we compared the performance of each risk score to age and then their modified versions by removing the age component from the risk scores.

### Role of cardiometabolic disease (CHD, stroke, diabetes) in the association between risk scores and dementia

In participants free of cardiometabolic disease at the assessment of risk scores, we examined the role of cardiometabolic disease over the follow-up in the association between risk scores and incidence of dementia using multistate models (Additional file 1: Fig. S2). These models allow simultaneous estimation of the risk associated with the risk scores (CAIDE, FRS, FINDRISC) in three transitions (or change in health states) over the follow-up: (1) from a healthy state to incidence of cardiometabolic disease, (2) from cardiometabolic disease over the follow-up to incidence of dementia, and (3) from a healthy state to incidence of dementia in those free of cardiometabolic disease over the follow-up. Participants who died over the follow-up were censored at date of death in order to take the competing risk of death into account [27]. Age was used as the timescale, and analyses were undertaken using R (mstate). These analyses were undertaken using risk scores from 1991 to 1993 and at ages 55, 60, and 65.

### Sensitivity analysis

Two sets of analyses were carried out. As information on dementia subtype was not available for all cases, we used data on the history of cardiovascular disease (myocardial infarction or stroke) over the follow-up to create a proxy indicator for Alzheimer’s disease dementia defined as dementia without a history of cardiovascular disease. We also undertook analysis using Fine and Gray subdistribution hazard models to assess whether the results differed using an alternative method of taking the competing risk of mortality. This method is recommended when the focus is on quantifying an individual’s absolute risk [22]; although this was not our focus, we examined whether results using this approach were broadly consistent with the main findings.

## Results

Of the 10,308 participants recruited to the Whitehall II study in 1985–1988, a total of 8814 participated at the 1991–1993 wave (Additional file 1: Fig. S3). The analyses using risk scores from 1991 to 1993 were based on 7553 participants, followed for incidence of dementia. Table 1 presents characteristics of this study population in 1991–1993, mean age of participants was 50 (range 39 to 63) years, and 318 cases of dementia recorded over a mean follow-up of 23.5 (SD = 4.0) years. As expected, there was accelerated cognitive decline in the years leading to dementia diagnosis (Additional file 1: Fig. S4), supporting the validity of dementia ascertainment.

**Table 1:** Characteristics of participants in 1991–1993 as a function of dementia status at the end of the follow-up in March 2017

	All participants	No dementia diagnosed	Dementia diagnosed	*p*^a^
**Risk factors in CAIDE**	**7553**	**7235**	**318**	
Age (years)				< 0.001
< 47	2871 (38.0%)	2849 (39.4%)	22 (6.9%)	
47–53	2069 (27.4%)	2012 (27.8%)	57 (17.9%)	
> 53	2613 (34.6%)	2374 (32.8%)	239 (75.2%)	
Sex				< 0.001
Male	5230 (69.2%)	5043 (69.7%)	187 (58.8%)	
Female	2323 (30.8%)	2192 (30.3%)	131 (41.2%)	
Education (years)				< 0.001
≥ 10	4076 (54.0%)	3944 (54.5%)	132 (41.5%)	
7–9	2643 (35.0%)	2518 (34.8%)	125 (39.3%)	
0–6	834 (11.0%)	773 (10.7%)	61 (19.2%)	
Vigorous physical activity^b^				0.030
Yes	1937 (25.6%)	1872 (25.9%)	65 (20.4%)	
No	5616 (74.4%)	5363 (74.1%)	253 (79.6%)	
BMI (kg/m^2^)				0.002
≤ 30	6837 (90.5%)	6565 (90.7%)	272 (85.5%)	
> 30	716 (9.5%)	670 (9.3%)	46 (14.5%)	
SBP (mmHg)				< 0.001
≤ 140	6951 (92.0%)	6674 (92.2%)	277 (87.1%)	
> 140	602 (8.0%)	561 (7.8%)	41 (12.9%)	
Total cholesterol (mmol/L)				0.003
≤ 6.5	4019 (53.2%)	3876 (53.6%)	143 (45.0%)	
> 6.5	3534 (46.8%)	3359 (46.4%)	175 (55.0%)	
**Risk factors in FRS or FINDRISC**				
Current smokers				0.230
No	6535 (86.5%)	6267 (86.6%)	268 (84.3%)	
Yes	1018 (13.5%)	968 (13.4%)	50 (15.7%)	
Daily fruit or vegetable consumption				0.440
Yes	4621 (61.2%)	4433 (61.3%)	188 (59.1%)	
No	2932 (38.8%)	2802 (38.7%)	130 (40.9%)	
Diabetes				< 0.001
No	7385 (97.8%)	7083 (97.9%)	302 (95.0%)	
Yes	168 (2.2%)	152 (2.1%)	16 (5.0%)	
Waist circumference (cm)				< 0.001
Men < 94, women< 80	5715 (75.7%)	5500 (76.0%)	215 (67.6%)	
Men 94 to < 102 women 80 to < 88	1156 (15.3%)	1101 (15.2%)	55 (17.3%)	
Men ≥ 102, women ≥ 88	682 (9.0%)	634 (8.8%)	48 (15.1%)	
HDL cholesterol (mmol/L)				0.560
≥ 0.9	7066 (93.6%)	6766 (93.5%)	300 (94.3%)	
< 0.9	487 (6.4%)	469 (6.5%)	18 (5.7%)	

*CAIDE* Cardiovascular Risk Factors, Aging, and Incidence of Dementia, *FRS* Framingham cardiovascular Risk Score, *FINDRISC* Finnish Diabetes Risk Score

^a^*p* value for chi-squared statistics

^b^Physical activity causing sweating ≥ 2 times/week for a total weekly duration ≥ 1 h

The CAIDE study used logistic regression to calculate predictive performance as data on dementia status were not available throughout the follow-up. This was not the case in our study as incidence of dementia and date of death (to take competing risk into account) were available over the entire follow-up, leading us to use survival analyses. The C-statistic obtained using logistic regression and a 20-year follow-up in our study (0.80; 95% CI 0.78, 0.82) is comparable to that in the CAIDE study (0.77; 95% CI 0.71, 0.83).

**Fig. 1:**
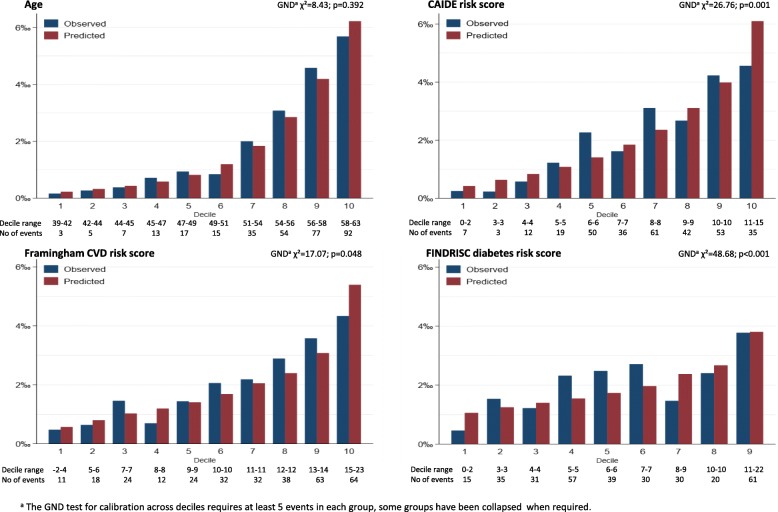
Observed and predicted rates of dementia per 1000 person/years (calibration-in-the-large) as a function of deciles predictors (age, CAIDE, FRS, and FINDRISC)

The predictive performance of all three risk scores, drawn from baseline in 1991–1993, for dementia is presented in Table 2. FINDRISC had the weakest association with dementia (HR = 1.52; 95% CI 1.38, 1.67), and C-statistic was also lower than CAIDE (0.630 compared to 0.714, *p* < 0.001, Table 2). CAIDE and FRS had similar discrimination (C-statistic of 0.714 and 0.719, respectively, *p* = 0.727) but CAIDE had a slightly better fit (Δ_AIC_ = 3.3). Calibration, reflecting the agreement between observed outcomes and prediction, shows age on its own to do better than the risk scores (Fig. 1); GND values suggest poor calibration for CAIDE and FINDRISC.

**Table 2:** Association of standardized risk scores (CAIDE, FRS, and FINDRISC) with incidence of dementia

	HR (95% CI)	*R*^2^ (95% CI)	C-statistic (95% CI)	*p*^e^	AIC	Δ_AIC_
**Risk scores assessed in 1991–1993**^**a**^**, age 39 to 63 years (mean follow-up 23.5 years; 318 cases of dementia in 7553 participants)**						
CAIDE	2.24 (1.98, 2.52)	32.5 (25.5, 39.4)	0.714 (0.690, 0.739)	Ref.	5403.2	Ref.
FRS	2.08 (1.87, 2.31)	32.0 (24.9, 39.5)	0.719 (0.693, 0.745)	0.727	5406.5	3.3
FINDRISC	1.52 (1.38, 1.67)	12.5 (7.47, 19.3)	0.630 (0.602, 0.659)	< 0.001	5521.2	118.0
**Analysis stratified by baseline age**						
**Risk scores assessed at 55 years**^**b**^**(mean follow-up 17.8 years; 267 cases of dementia in 6773 participants)**						
CAIDE	1.22 (1.09, 1.38)	2.53 (0.31, 6.55)	0.552 (0.514, 0.590)	Ref.	4264.3	Ref.
FRS	1.43 (1.26, 1.61)	6.97 (2.77, 13.0)	0.584 (0.547, 0.621)	0.136	4244.5	− 19.8
FINDRISC	1.25 (1.10, 1.41)	2.72 (0.35, 6.73)	0.560 (0.522, 0.599)	0.692	4263.5	− 0.8
**Risk scores assessed at 60 years**^**c**^**(mean follow-up 14 years; 301 cases of dementia in 7008 participants)**						
CAIDE	1.03 (0.92, 1.16)	0.06 (0.00, 1.60)	0.496 (0.455, 0.537)	Ref.	4636.3	Ref.
FRS	1.23 (1.10, 1.38)	2.57 (0.52, 6.38)	0.563 (0.526, 0.600)	0.003	4623.8	− 12.5
FINDRISC	1.20 (1.07, 1.36)	1.75 (0.08, 5.32)	0.531 (0.489, 0.572)	0.131	4627.9	− 8.4
**Risk scores assessed at 65 years**^**d**^**(mean follow-up 9.6 years; 267 cases of dementia in 6455 participants)**						
CAIDE	1.05 (0.93, 1.18)	0.13 (0.00, 1.85)	0.504 (0.463, 0.546)	Ref.	3984.9	Ref.
FRS	1.13 (1.01, 1.27)	1.06 (0.01, 4.22)	0.533 (0.493, 0.574)	0.240	3980.8	− 4.1
FINDRISC	1.13 (1.00, 1.28)	0.81 (0.01, 3.68)	0.517 (0.476, 0.558)	0.613	3981.9	− 3.0

*CAIDE* Cardiovascular Risk Factors, Aging, and Incidence of Dementia, *FRS* Framingham cardiovascular Risk Score, *FINDRISC* Finnish Diabetes Risk Score, *HR* hazard ratio, *CI* confidence interval, *R*^*2*^ Royston’s *R*^2^, *C-index* Harrell’s C-index, *AIC* Akaike information criterion

^a^1991–1993: 1 SD corresponds to 2.9 points in CAIDE, 3.7 points for men and 4.2 points for women in FRS, and 3.3 points for men and 3.9 points for women in FINDRISC

^b^Age 55: 1 SD corresponds to 1.9 points in CAIDE, 3.4 points for men and 3.6 points for women in FRS, and 3.6 points for men and 4.0 points for women in FINDRISC

^c^Age 60: 1 SD corresponds to 1.9 points in the CAIDE, 3.1 points for men and 3.6 points for women in FRS, and 3.8 points for men and 4.1 points for women in FINDRISC

^d^Age 65: 1 SD corresponds to 1.9 points in the CAIDE, 2.9 points for men and 3.6 points for women in FRS, and 4.0 points for men and 4.4 points for women in FINDRISC

^e^*p* values for difference in C-statistic

Analyses stratified by age were based on risk scores for each participant using data from waves closest to when participants were 55 (mean = 55.6; SD = 2.3), 60 (mean = 59.9; SD = 2.0), and 65 (mean = 64.6; SD = 2.1) years; exact age was used in the calculation of the risk scores. The performance indicators for the three risk scores were considerably poorer in these analyses (Table 2), with poor discrimination (C-statistic lower than 0.60) and variance explained (*R*^2^ less than 10%). CAIDE was associated with dementia when assessed at age 55 (HR = 1.22; 95% CI 1.09, 1.38) but not at age 60 (HR = 1.03; 95% CI 0.92, 1.16) or 65 (HR = 1.05; 95% CI 0.93, 1.18). Although all scores performed poorly in these analyses stratified by age at risk assessment, FRS and FINDRISC had better predictive ability than the CAIDE as assessed by *R*^2^ (CAIDE always the lowest), C-statistic (CAIDE always the lowest, albeit not significantly), and AIC (Δ_AIC_ > 3). Further complimentary analyses on the role of age show that age on its own, ranging from 39 to 63 years, had the strongest association with dementia and better predictive performance than all three risk scores (Additional file 1: Table S5).

In sensitivity analyses, we repeated these analyses using a proxy for Alzheimer’s disease dementia (dementia cases without a history of cardiovascular disease); findings were broadly similar to that in the main analyses (Additional file 1: Table S6). Analysis using subdistribution hazard models for competing risk of death were also similar to those obtained using cause-specific hazard models (Additional file 1: Table S7).

Table 3 presents results from the multistate models. In analyses using risk scores from 1991 to 1993, all three risk scores were associated with higher risk of dementia in those who remained free of cardiometabolic disease over the follow-up (transition “healthy to dementia”, Table 3). In analyses stratified by age at risk factor assessment, only FRS at age 55 was associated with risk of dementia in those free of clinical cardiometabolic disease over the mean follow-up of 17.8 years (HR = 1.33; 95% CI 1.11, 1.60). As expected, all three risk scores were associated with incidence of cardiometabolic disease, irrespective of the age at which risk factors were assessed (transition “healthy to cardiometabolic disease”, Table 3).

**Table 3:** Multistate models for the transitions from a healthy state to cardiometabolic disease and dementia

Transitions	HR (95% CI)
**Risk scores assessed in 1991–1993, age 39 to 63 years**^**a**^	
**CAIDE**	
Healthy to incident cardiometabolic disease	1.54 (1.47, 1.61)
Cardiometabolic disease to dementia	2.10 (1.69, 2.59)
Healthy to dementia	2.13 (1.81, 2.50)
**FRS**	
Healthy to incident cardiometabolic disease	1.76 (1.69, 1.84)
Cardiometabolic disease to dementia	1.81 (1.50, 2.20)
Healthy to dementia	2.07 (1.78, 2.41)
**FINDRISC**	
Healthy to incident cardiometabolic disease	1.62 (1.56, 1.68)
Cardiometabolic disease to dementia	1.21 (1.02, 1.44)
Healthy to dementia	1.46 (1.26, 1.69)
**Analysis stratified by baseline age**	
**Risk scores assessed at 55 years**^**b**^	
**CAIDE**	
Healthy to incident cardiometabolic disease	1.33 (1.27, 1.39)
Cardiometabolic disease to dementia	1.22 (1.00, 1.49)
Healthy to dementia	1.13 (0.97, 1.33)
**FRS**	
Healthy to incident cardiometabolic disease	1.54 (1.47, 1.63)
Cardiometabolic disease to dementia	1.25 (1.00, 1.56)
Healthy to dementia	1.33 (1.11, 1.60)
**FINDRISC**	
Healthy to incident cardiometabolic disease	1.58 (1.50, 1.66)
Cardiometabolic disease to dementia	1.07 (0.86, 1.32)
Healthy to dementia	1.16 (0.96, 1.41)
**Risk scores assessed at 60 years**^**c**^	
**CAIDE**	
Healthy to incident cardiometabolic disease	1.27 (1.20, 1.33)
Cardiometabolic disease to dementia	0.98 (0.81, 1.19)
Healthy to dementia	0.97 (0.82, 1.13)
**FRS**	
Healthy to incident cardiometabolic disease	1.47 (1.39, 1.55)
Cardiometabolic disease to dementia	1.13 (0.91, 1.40)
Healthy to dementia	1.13 (0.95, 1.33)
**FINDRISC**	
Healthy to incident cardiometabolic disease	1.56 (1.48, 1.65)
Cardiometabolic disease to dementia	0.89 (0.70, 1.12)
Healthy to dementia	1.06 (0.87, 1.29)
**Risk scores assessed at 65 years**^**d**^	
**CAIDE**	
Healthy to incident cardiometabolic disease	1.24 (1.17, 1.32)
Cardiometabolic disease to dementia	0.92 (0.73, 1.17)
Healthy to dementia	1.00 (0.85, 1.18)
**FRS**	
Healthy to incident cardiometabolic disease	1.40 (1.31, 1.50)
Cardiometabolic disease to dementia	1.07 (0.84, 1.36)
Healthy to dementia	1.00 (0.85, 1.17)
**FINDRISC**	
Healthy to incident cardiometabolic disease	1.56 (1.45, 1.68)
Cardiometabolic disease to dementia	0.82 (0.62, 1.09)
Healthy to dementia	0.89 (0.72, 1.10)

*CAIDE* Cardiovascular Risk Factors, Aging, and Incidence of Dementia, *FRS*: Framingham cardiovascular Risk Score, *FINDRISC* Finnish Diabetes Risk Score

^a^*N* overall = 7159 free of cardiometabolic disease and dementia; *N* = 2105 with incident cardiometabolic disease, 113 of whom developed dementia subsequently; *N* = 5054 free of cardiometabolic disease over the follow-up, 175 developed dementia

^b^*N* overall = 6162 free of cardiometabolic disease and dementia; *N* = 1637 with incident cardiometabolic disease, 92 of whom developed dementia subsequently; *N* = 4525 free of cardiometabolic disease over the follow-up, 148 developed dementia

^c^*N* overall = 6074 free of cardiometabolic disease and dementia; *N* = 1427 with incident cardiometabolic disease, 97 of whom developed dementia subsequently; *N* = 4647 free of cardiometabolic disease over the follow-up, 162 developed dementia

^d^*N* overall = 5242 free of cardiometabolic disease and dementia; *N* = 931 with incident cardiometabolic disease, 68 of whom developed dementia subsequently; *N* = 4311 free of cardiometabolic disease over the follow-up, 144 developed dementia

## Discussion

The long course of dementia makes midlife an important target for dementia prevention. Given the multifactorial etiology of dementia, risk scores represent an effective prevention tool but CAIDE is the only existing midlife risk score constructed specifically for dementia. We compared its predictive performance for dementia to risk scores developed for cardiovascular disease (FRS) and diabetes (FINDRISC) and found it not to be better at predicting dementia. We also found all three risk scores to have poor discrimination (C-statistic < 0.60) for dementia when the effect of age was neutralized by assessing risk factors at ages 55, 60, and 65 years. Finally, analyses using multistate models showed only the Framingham cardiovascular score at age 55 to be associated with risk of dementia in those who remained free of cardiovascular disease and diabetes until dementia diagnosis.

Most existing risk scores for dementia are based on predictors assessed at older ages, with reviews concluding that their predictive accuracy is poor [28–30]. We did not assess the predictive performance of risk scores constructed for use at older ages; our focus was midlife as the pathophysiological processes underlying dementia unfold over many years, perhaps decades [31], making it important to consider the age at assessment of risk factors. CAIDE, based on midlife predictors, was reported as having a C-statistic of 0.77 in the derivation cohort [12]. A study using Kaiser Permanente data on adults aged 40–55 years at risk factor assessment, followed for a mean 36.1 years, reported a C-statistic of 0.75 [32] for CIADE but analyses of individual components revealed only age and sex of the seven components in CAIDE to be associated with risk of dementia. Given that 4 of the 15 points in the score are due to age (age < 47 scored 0, 47–45 scored 3, and > 53 years scored 4), it is likely to be an important driver of its predictive ability. In the Rotterdam study where all participants were older than 53 at the start of follow-up (hence, had a score of 4 for age), the C-statistic for CAIDE after a 15-year follow-up was only 0.55 (95% CI 0.53, 0.58) [33]. In our data using risk factors from 1991 to 1993, the C-statistic was 0.71 but it fell to 0.55 (95% CI 0.51, 0.59) when risk factors for all participants were assessed at age 55, matching results obtained in the Rotterdam study. Thus, the predictive ability of the CAIDE dementia risk score beyond age was poor at best.

As cardiometabolic risk factors feature prominently in dementia prevention guidelines [2], we examined whether risk profiles developed for cardiovascular disease and diabetes are useful in predicting dementia. Our results do not provide strong evidence for their utility in predicting dementia with the caveat that risk factors included in these algorithms and their categorization were not optimized for dementia prediction. Two results are particularly noteworthy. One, analysis using risk scores constructed at ages 55, 60, and 65 years to remove the effect of age showed the cardiovascular disease and diabetes risk scores to be associated (HR had *p* < 0.05) with late-life dementia in contrast to the CAIDE where associations were found only when risk factors were assessed at age 55. Two, only FRS at age 55 was associated with risk of dementia in persons free from clinical cardiometabolic disease at dementia diagnosis.

The long preclinical phase of dementia and the absence of effective disease modification have led to an interest in prevention. Furthermore, several risk factors have an age-dependent association with dementia, particularly cardiovascular risk factors where the risk of dementia is shaped by mid- rather than later-life exposure. This is reflected in our findings for all three risk scores as their predictive performance is systematically better when assessed at age 55 than at age 65 years. The risk score approach can be useful for multifactorial conditions, as demonstrated by the example of cardiovascular disease [34, 35]. Better understanding of risk factors has led to the development of both therapeutic strategies and public health campaigns that targeted major risk factors, leading to declines in cardiovascular disease. A similar approach for dementia would be valuable, but it requires consideration of a larger set of predictors (e.g., smoking, cardiovascular disease, glucose, insulin, and inflammatory markers) with careful categorization to best reflect the continuum of risk. For risk factors such as systolic blood pressure, there is now evidence that the 140-mmHg threshold might not adequately capture risk [10]. Cardiovascular risk scores were elaborated and continue to be modified, to better take into account key risk factors along with appropriate categorization of risk factors to improve the predictive ability of risk scores. A similar effort is now needed for dementia prevention and assessment of the predictive ability of existing risk scores is the first step in that process.

Our findings need to be considered in light of the study’s strengths and limitations. Strengths include the longitudinal design and repeat risk factor assessments allowing age-specific analyses of dementia prediction and the relatively large population-based sample with the main analysis on 318 cases of dementia compared to 61 in the study population used to develop the CAIDE score. A limitation of the study is the ascertainment of dementia being based on linkage to electronic health records. In the Mayo Clinic Study of Aging and the Adult Changes study, a comparison of passive case finding to active approach showed the passive approach to have high specificity, approximately 70% sensitivity, and to miss mostly milder cases of dementia [36]. A similar pattern is likely in our study as health coverage is universal in the UK, and electronic health records have been shown to be reliable for the ascertainment of dementia status [37]. As dementia ascertainment in our study is independent from the assessment of risk factors, major bias is unlikely. Furthermore, we were able to undertake analyses on everyone with data on risk factors rather than only those who were alive 20 years later and participated in an in-person assessment of dementia status. Finally, we were unable to examine the subtypes of dementia due to small numbers. However, our analysis of dementia without a history of cardiovascular disease as a proxy for Alzheimer’s disease suggests that the findings are likely to be generalizable to all major types of dementia.

## Conclusions

Dementia is a worldwide health, economic and social-care priority. The latest systematic review of global prevalence estimates the number of people living with dementia at 46.8 million with this number expected to double every 20 years until 2050 [38]. Even a 1-year delay in dementia onset is projected to lead to 9.2 million fewer cases worldwide by 2050 [39]. However, the manner in which this can be achieved remains unclear. Previous research shows late-life risk scores not to be useful for dementia prediction. Our analyses of CAIDE, a midlife dementia risk score, and risk scores for cardiovascular disease and diabetes show the FRS in midlife to predict dementia as well as the CAIDE risk score, its predictive value being also evident among individuals who did not develop cardiometabolic events. It is noteworthy that much of the predictive ability of these risk scores was dependent on age. These findings highlight the need for better tools to identify individuals who could benefit most from more aggressive midlife prevention measures.

## Supplementary information

**Additional file 1.** Methodological details and complimentary results.

## Data Availability

The dataset used is available via the Whitehall II study data sharing mechanism, https://www.ucl.ac.uk/epidemiology-health-care/research/epidemiology-and-public-health/research/whitehall-ii/data-sharing

## Abbreviations

AIC: Akaike information criterion
BMI: Body mass index
CAIDE: Cardiovascular Risk Factors, Aging, and Incidence of Dementia
CHD: Coronary heart disease
FINDRISC: Finnish Diabetes Risk Score
FRS: Framingham cardiovascular Risk Score
HES: Hospital episode statistics
ICD: International Classification of Disease
MMSE: Mini-Mental State Examination
NHS: National Health Service
STROBE: STrengthening the Reporting of OBservational studies in Epidemiology
