# Supplementary material for: Risk prediction models for dementia: role of age and cardiometabolic risk factors
Source: BMC Med. 2020 May 19;18:107. doi: 10.1186/s12916-020-01578-x (PMC7236124; doi:10.1186/s12916-020-01578-x)
Supplement: Supplementary file 1 — Additional file 1. Methodological details and complimentary results. [file 12916_2020_1578_MOESM1_ESM.docx]

**Risk prediction models for dementia: role of age and cardiometabolic risk factors**

**Additional file 1: Methodological details and complimentary results.**

Contents

[Table S1. Scoring of the Cardiovascular Risk Factors, Aging, and Incidence of Dementia (CAIDE) risk score. 2](#_Toc35505226)

[Table S2. Scoring of the Framingham cardiovascular Risk Score (FRS). 3](#_Toc35505227)

[Table S3. Scoring of the Finnish Diabetes Risk Score (FINDRISC). 4](#_Toc35505228)

[Table S4. TRIPOD checklist 5](#_Toc35505229)

[Figure S1. Association of standardized predictors (CAIDE, FRS, and FINDRISC) assessed at age 55 with incidence of dementia^a^ (Panel A) and their distribution (Panel B). 6](#_Toc35505230)

[Figure S2. Schematic representation of role of risk scores in transitions examined using multistate models. 7](#_Toc35505231)

[Figure S3. Flowchart of sample selection for analysis on dementia with assessment of risk scores in 1991-1993. 8](file:///C:\Users\Archana%20Singh-Manoux\Desktop\Additional%20file%201_R3.docx#_Toc35505232)

[Figure S4. Trajectory of global cognitive score ^a^ in dementia cases in the years leading to dementia diagnosis and dementia free participants until end of follow-up. 9](#_Toc35505233)

[Table S5. Association of age and standardized risk scores (CAIDE, FRS, and FINDRISC) assessed in 1991-1993, with and without age, with incidence of dementia; mean follow-up 23.5 years; 318 cases of dementia in 7,553 participants. 10](#_Toc35505234)

[Table S6. Association of standardized risk scores (CAIDE, FRS, and FINDRISC) assessed at age 55, 60, and 65 with incidence of dementia without history of cardiovascular disease. 11](#_Toc35505235)

[Table S7. Association of standardized risk scores (CAIDE, FRS, and FINDRISC) with incidence of dementia using cause-specific and subdistribution hazard to account for competing risk of death. 12](#_Toc35505236)

| Table S1. Scoring of the Cardiovascular Risk Factors, Aging, and Incidence of Dementia (CAIDE) risk score. | | | | | | | |
| --- | --- | --- | --- | --- | --- | --- | --- |
| **Points** | **Age (years)** | **Education (years)** | **Sex** | **Physical activity^†^** | **SBP (mmHg)** | **BMI (kg/m²)** | **Total cholesterol (mmol/L)** |
| 0 | <47 | ≥10 | Women | Active | ≤140 | ≤30 | ≤6.5 |
| 1 |  |  | Men | Inactive |  |  |  |
| 2 |  | 7-9 |  |  | >140 | >30 | >6.5 |
| 3 | 47-53 | 0-6 |  |  |  |  |  |
| 4 | >53 |  |  |  |  |  |  |
| SBP=systolic blood pressure, BMI=body-mass index  ^†^Physical activity causing sweating ≥ 2/week for a total weekly duration of one hour or more. | | | | | | | |

| Table S2. Scoring of the Framingham cardiovascular Risk Score (FRS). | | | | | | | |
| --- | --- | --- | --- | --- | --- | --- | --- |
| **Points** | **Age (years)** | **Smoker** | **Diabetic** | **SBP not treated (mmHg)** | **SBP treated (mmHg)** | **HDL (mg/dL)** | **Total cholesterol (mg/dL)** |
| **Women** | | | | | | | |
| -3 |  |  |  | <120 |  |  |  |
| -2 |  |  |  |  |  | ≥60 |  |
| -1 |  |  |  |  | <120 | 50-59 |  |
| 0 | 30-34 | No | No | 120-129 |  | 45-49 | <160 |
| 1 |  |  |  | 130-139 |  | 35-44 | 160-199 |
| 2 | 35-39 |  |  | 140-149 | 120-129 | <35 |  |
| 3 |  | Yes |  |  | 130-139 |  | 200-239 |
| 4 | 40-44 |  | Yes | 150-159 |  |  | 240-279 |
| 5 | 45-49 |  |  | ≥160 | 140-149 |  | ≥280 |
| 6 |  |  |  |  | 150-159 |  |  |
| 7 | 50-54 |  |  |  | ≥160 |  |  |
| 8 | 55-59 |  |  |  |  |  |  |
| 9 | 60-64 |  |  |  |  |  |  |
| 10 | 65-69 |  |  |  |  |  |  |
| 11 | 70-74 |  |  |  |  |  |  |
| 12 | ≥75 |  |  |  |  |  |  |
| **Men** | | | | | | | |
| -2 |  |  |  | <120 |  | ≥60 |  |
| -1 |  |  |  |  |  | 50-59 |  |
| 0 | 30-34 | No | No | 120-129 | <120 | 45-49 | <160 |
| 1 |  |  |  | 130-139 |  | 35-44 | 160-199 |
| 2 | 35-39 |  |  | 140-159 | 120-129 | <35 | 200-239 |
| 3 |  |  | Yes | ≥160 | 130-139 |  | 240-279 |
| 4 |  | Yes |  |  | 140-159 |  | ≥280 |
| 5 | 40-44 |  |  |  | ≥160 |  |  |
| 6 | 45-49 |  |  |  |  |  |  |
| 8 | 50-54 |  |  |  |  |  |  |
| 10 | 55-59 |  |  |  |  |  |  |
| 11 | 60-64 |  |  |  |  |  |  |
| 12 | 65-69 |  |  |  |  |  |  |
| 14 | 70-74 |  |  |  |  |  |  |
| 15 | ≥75 |  |  |  |  |  |  |

SBP=systolic blood pressure, HDL=high-density lipoprotein

| Table S3. Scoring of the Finnish Diabetes Risk Score (FINDRISC). | | | | | | | | | |
| --- | --- | --- | --- | --- | --- | --- | --- | --- | --- |
| **Points** | **Age (years)** | **Family diabetes** | **Daily fruit/veg.** | **Physical activity^†^** | **HBP medication** | **History of HBG** | **BMI (kg/m²)** | **Waist circumference (cm)** | |
|  |  |  |  |  |  |  |  | **Women** | **Men** |
| 0 | <45 | No | Yes | Yes | Never | No | ≤25 | <80 | <94 |
| 1 |  |  | No |  |  |  | 25-30 |  |  |
| 2 | 45-54 |  |  | No | At least once |  |  |  |  |
| 3 | 55-64 | 2^nd^ deg. |  |  |  |  | >30 | 80-88 | 94-102 |
| 4 | ≥65 |  |  |  |  |  |  | ≥88 | ≥102 |
| 5 |  | 1^st^ deg. |  |  |  | Yes |  |  |  |
| HBP=High blood pressure, HBG=high blood glucose, BMI=body-mass index  ^†^Moderate to vigorous physical activity ≥30min/day, as defined by the 2011 Compendium of Physical Activities | | | | | | | | | |

# Table S4. TRIPOD checklist

| **Section/Topic** | **Item** | **Checklist Item** | **Page** |
| --- | --- | --- | --- |
| **Title and abstract** | | | |
| Title | 1 | Identify the study as developing and/or validating a multivariable prediction model, the target population, and the outcome to be predicted. | 1 |
| Abstract | 2 | Provide a summary of objectives, study design, setting, participants, sample size, predictors, outcome, statistical analysis, results, and conclusions. | 2 |
| **Introduction** | | | |
| Background and objectives | 3a | Explain the medical context (including whether diagnostic or prognostic) and rationale for developing or validating the multivariable prediction model, including references to existing models. | 4 |
|  | 3b | Specify the objectives, including whether the study describes the development or validation of the model or both. | 4,5 |
| **Methods** | | | |
| Source of data | 4a | Describe the study design or source of data (e.g., randomized trial, cohort, or registry data), separately for the development and validation data sets, if applicable. | 5 |
|  | 4b | Specify the key study dates, including start of accrual; end of accrual; and, if applicable, end of follow-up. | 5 |
| Participants | 5a | Specify key elements of the study setting (e.g., primary care, secondary care, general population) including number and location of centres. | 5 |
|  | 5b | Describe eligibility criteria for participants. | 5 |
|  | 5c | Give details of treatments received, if relevant. | 5-7 |
| Outcome | 6a | Clearly define the outcome that is predicted by the prediction model, including how and when assessed. | 7 |
|  | 6b | Report any actions to blind assessment of the outcome to be predicted. | NA |
| Predictors | 7a | Clearly define all predictors used in developing or validating the multivariable prediction model, including how and when they were measured. | 5-7 |
|  | 7b | Report any actions to blind assessment of predictors for the outcome and other predictors. | NA |
| Sample size | 8 | Explain how the study size was arrived at. | Fig. S3 |
| Missing data | 9 | Describe how missing data were handled (e.g., complete-case analysis, single imputation, multiple imputation) with details of any imputation method. | Fig. S3 |
| Statistical analysis methods | 10c | For validation, describe how the predictions were calculated. | 7-9 |
|  | 10d | Specify all measures used to assess model performance and, if relevant, to compare multiple models. | 7-9 |
|  | 10e | Describe any model updating (e.g., recalibration) arising from the validation, if done. | NA |
| Risk groups | 11 | Provide details on how risk groups were created, if done. | 7-9 |
| Development vs. validation | 12 | For validation, identify any differences from the development data in setting, eligibility criteria, outcome, and predictors. | 10 |
| **Results** | | | |
| Participants | 13a | Describe the flow of participants through the study, including the number of participants with and without the outcome and, if applicable, a summary of the follow-up time. A diagram may be helpful. | Fig. S3 |
|  | 13b | Describe the characteristics of the participants (basic demographics, clinical features, available predictors), including the number of participants with missing data for predictors and outcome. | Table 1 |
|  | 13c | For validation, show a comparison with the development data of the distribution of important variables (demographics, predictors and outcome). | Table 1 |
| Model performance | 16 | Report performance measures (with CIs) for the prediction model. | Tables 2, 3 |
| Model-updating | 17 | If done, report the results from any model updating (i.e., model specification, model performance). | NA |
| **Discussion** | | | |
| Limitations | 18 | Discuss any limitations of the study (such as nonrepresentative sample, few events per predictor, missing data). | 13-14 |
| Interpretation | 19a | For validation, discuss the results with reference to performance in the development data, and any other validation data. | 12 |
|  | 19b | Give an overall interpretation of the results, considering objectives, limitations, results from similar studies, and other relevant evidence. | 11-12 |
| Implications | 20 | Discuss the potential clinical use of the model and implications for future research. | 14-15 |
| **Other information** | | | |
| Supplementary information | 21 | Provide information about the availability of supplementary resources, such as study protocol, Web calculator, and data sets. | Additional file 1 |
| Funding | 22 | Give the source of funding and the role of the funders for the present study. | 16 |

# Figure S1. Association of standardized predictors (CAIDE, FRS, and FINDRISC) assessed at age 55 with incidence of dementia^a^ (Panel A) and their distribution (Panel B).

**Panel A Panel B**

CAIDE=Cardiovascular Risk Factors, Aging, and Incidence of Dementia; FRS: Framingham cardiovascular Risk Score; FINDRISC: Finnish Diabetes Risk Score.

**^a^**Hazard ratio estimated from restricted cubic spline (4 knots) and their 95% confidence interval 95%, scale for the predictors was reduced to 2 standard deviation over the mean.

# Figure S2. Schematic representation of role of risk scores in transitions examined using multistate models.

**Transition 1: Healthy to Incident Cardiometabolic Disease**

Incident cardiometabolic disease

Start of follow-up

**Transition 2 : Cardiometabolic disease to Dementia**

**Transition 3: Healthy to Dementia**

Dementia

**1985-1988, N=10,308** Baseline of the Whitehall II study

**1991-1993, N=8,814** free of dementia

1,494 excluded participants
 No response/withdrawal before 1991-1993 (n=1,369)
 Died before 1991-1993 (n=125)

**Analysis: Dementia**

Follow-up form 1991-1993 to

31^st^ March 2017

**N Dementia/Total=318/7553**

**N Dementia/Total=318/7553**

1,261 missing data on at least one score
 CAIDE (n=947)
 Framingham CVD (n=1,008)
 FINDRISC (n=1,151)

# Figure S3. Flowchart of sample selection for analysis on dementia with assessment of risk scores in 1991-1993.

# Figure S4. Trajectory of global cognitive score ^a^ in dementia cases in the years leading to dementia diagnosis and dementia free participants until end of follow-up.

|  | **Number of observations in the analysis** | | | | | |
| --- | --- | --- | --- | --- | --- | --- |
| **Years** | | **-20 to -16** | **-16 to -12** | **-12 to-8** | **-8 to-4** | **-4 to 0** |
| **Dementia free (N=7237)** | | 5136 | 5693 | 5130 | 5788 | 5768 |
| **Dementia cases (N=291)** | | 88 | 125 | 167 | 177 | 145 |

^a^ Composed of tests of memory, reasoning, phonemic and semantic fluency administered to the participants in 1997, 2003, 2007, 2012, and 2015.

# Table S5. Association of age and standardized risk scores (CAIDE, FRS, and FINDRISC) assessed in 1991-1993, with and without age, with incidence of dementia; mean follow-up 23.5 years; 318 cases of dementia in 7,553 participants.

|  | **HR (95% CI)** |  | **R² (95% CI)** |  | **C-statistic (95% CI)** | **p** | **p^d^** |  | **AIC** |
| --- | --- | --- | --- | --- | --- | --- | --- | --- | --- |
| Age (39-63 years)^a^ | 3.09 (2.70, 3.55) |  | 52.7 (45.6, 59.9) |  | 0.781 (0.758, 0.804) | Ref. |  |  | 5257.0 |
|  |  |  |  |  |  |  |  |  |  |
| **Risk scores** |  |  |  |  |  |  |  |  |  |
| CAIDE**^b^** | 2.24 (1.98, 2.52) |  | 32.5 (25.5, 39.4) |  | 0.714 (0.690, 0.739) | 0.001 | Ref. |  | 5403.2 |
| CAIDE (without age)**^c^** | 1.44 (1.30, 1.61) |  | 8.49 (4.21, 14.4) |  | 0.604 (0.573, 0.635) | 0.001 | 0.001 |  | 5543.4 |
| FRS**^b^** | 2.08 (1.87, 2.31) |  | 32.0 (24.9, 39.5) |  | 0.719 (0.693, 0.745) | 0.001 | Ref. |  | 5406.5 |
| FRS (without age) **^c^** | 1.43 (1.29, 1.59) |  | 8.11 (3.96, 14.1) |  | 0.600 (0.567, 0.632) | 0.001 | 0.001 |  | 5545.4 |
| FINDRISC**^b^** | 1.52 (1.38, 1.67) |  | 12.5 (7.47, 19.3) |  | 0.630 (0.602, 0.659) | 0.001 | Ref. |  | 5521.2 |
| FINDRISC (without age) **^c^** | 1.25 (1.14, 1.39) |  | 3.47 (0.83, 7.95) |  | 0.549 (0.516, 0.582) | 0.001 | 0.001 |  | 5570.3 |

CAIDE=Cardiovascular Risk Factors, Aging, and Incidence of Dementia; FRS: Framingham cardiovascular Risk Score; FINDRISC: Finnish Diabetes Risk Score.

HR: Hazard Ratio, CI: Confidence Interval, R²=Royston’s R², C-index=Harrell’s C-index, AIC=Akaike Information Criterion

^a^1 SD corresponds to 6.0 years.

^b^1 SD corresponds to 2.9 points in CAIDE, 3.7 points for men & 4.2 points for women in FRS, and 3.3 points for men & 3.9 points for women in FINDRISC.

^c^1 SD corresponds to 1.9 points in CAIDE without age, 2.8 points for men & 3.2 points for women in FRS without age, and 3.0 points for men & 3.5 points for women in FINDRISC without age.

^d^p-values for difference in C-statistic when risk score without the age component was compared to that with the age component.

# Table S6. Association of standardized risk scores (CAIDE, FRS, and FINDRISC) assessed at age 55, 60, and 65 with incidence of dementia without history of cardiovascular disease.

|  | **HR (95% CI)** | **R² (95% CI)** | **C-index (95% CI)** | | **p-value^e^** | **AIC** | **∆_AIC_** |
| --- | --- | --- | --- | --- | --- | --- | --- |
| **Risk scores assessed in 1991-1993,^a^ age 39 to 63 years (Mean follow-up 23.5 years; 249 cases of dementia in 7,484 participants)** | | | | | | | |
| CAIDE | 2.16 (1.88, 2.47) | 30.1 (22.5, 38.2) | | 0.706 (0.678, 0.734) | Ref. | 4243.0 | Ref. |
| FRS | 1.90 (1.69, 2.14) | 24.9 (17.7, 32.6) | | 0.698 (0.668, 0.728) | 0.556 | 4268.1 | 25.1 |
| FINDRISC | 1.49 (1.34, 1.66) | 11.4 (5.69, 18.5) | | 0.625 (0.593, 0.657) | <0.001 | 4329.0 | 86 |
| **Analysis stratified by baseline age** | | | | | | | |
| **Risk scores assessed at 55 years^b^ (Mean follow-up 17.8 years; 213 cases of dementia in 6,719 participants)** | | | | | | | |
| CAIDE | 1.21 (1.06, 1.38) | 2.17 (0.16, 6.66) | | 0.543 (0.502, 0.585) | Ref. | 3407.4 | Ref. |
| FRS | 1.29 (1.12, 1.49) | 3.57 (0.78, 8.82) | | 0.568 (0.526, 0.610) | 0.309 | 3402.5 | -4.9 |
| FINDRISC | 1.23 (1.07, 1.41) | 2.33 (0.21, 6.85) | | 0.557 (0.513, 0.600) | 0.584 | 3406.9 | -0.5 |
| **Risk scores assessed at 60 years^c^ (Mean follow-up 14.0 years; 237 cases of dementia in 6,944 participants)** | | | | | | | |
| CAIDE | 1.04 (0.91, 1.18) | 0.08 (0.00, 1.95) | | 0.500 (0.454, 0.546) | Ref. | 3657.9 | Ref. |
| FRS | 1.16 (1.02, 1.32) | 1.32 (0.04, 4.34) | | 0.558 (0.516, 0.599) | 0.024 | 3653.0 | -4.9 |
| FINDRISC | 1.21 (1.06, 1.39) | 1.90 (0.07, 6.21) | | 0.543 (0.497, 0.589) | 0.098 | 3650.7 | -7.2 |
| **Risk scores assessed at 65 years^d^ (Mean follow-up 9.5 years; 210 cases of dementia in 6,398 participants)** | | | | | | | |
| CAIDE | 1.04 (0.91, 1.19) | 0.11 (0.00, 1.95) | | 0.502 (0.455, 0.549) | Ref. | 3139.9 | Ref. |
| FRS | 1.12 (0.98, 1.27) | 0.82 (0.00, 3.97) | | 0.536 (0.492, 0.580) | 0.215 | 3137.4 | -2.5 |
| FINDRISC | 1.10 (0.96, 1.27) | 0.54 (0.00, 3.66) | | 0.507 (0.462, 0.553) | 0.851 | 3138.4 | -1.5 |

CAIDE=Cardiovascular Risk Factors, Aging, and Incidence of Dementia; FRS: Framingham cardiovascular Risk Score; FINDRISC: Finnish Diabetes Risk Score.

HR: Hazard Ratio, CI: Confidence Interval, R²=Royston’s R², C-index=Harrell’s C-index, AIC=Akaike Information Criterion

^a^**1991-1993**: 1 SD corresponds to 2.9 points in CAIDE, 3.7 points for men & 4.2 points for women in FRS, and 3.3 points for men & 3.9 points for women in FINDRISC.

**^b^Age 55**: 1 SD corresponds to 1.9 points in CAIDE, 3.4 points for men & 3.6 points for women in FRS, and 3.6 points for men & 4.0 points for women in FINDRISC.

**^c^** **Age 60**: 1 SD corresponds to 1.9 points in the CAIDE, 3.1 points for men & 3.6 points for women in FRS, and 3.8 points for men & 4.1 points for women in FINDRISC.

**^d^Age 65**: 1 SD corresponds to 1.9 points in the CAIDE, 2.9 points for men & 3.6 points for women in FRS, and 4.0 points for men & 4.4 points for women in FINDRISC.

^e^p-value for difference in C-statistic.

# Table S7. Association of standardized risk scores (CAIDE, FRS, and FINDRISC) with incidence of dementia using cause-specific and subdistribution hazard to account for competing risk of death.

|  | **Cause specific**  **HR (95% CI)** | **Subdistribution**  **HR (95% CI)** |  |
| --- | --- | --- | --- |
| **Risk scores assessed in 1991-1993,^a^ age 39 to 63 years (Mean follow-up 23.5 years; 249 cases of dementia & 1014 deaths in 7,484 participants)** | | | |
| CAIDE | 2.24 (1.98, 2.52) | 2.10 (1.89, 2.33) | |
| FRS | 2.08 (1.87, 2.31) | 1.90 (1.73, 2.08) | |
| FINDRISC | 1.52 (1.38, 1.67) | 1.45 (1.33, 1.59) | |
| **Analysis stratified by baseline age** | | | |
| **Risk scores assessed at 55 years^b^ (Mean follow-up 17.8 years; 213 cases of dementia & 1014 deaths & 821 deaths in 6,719 participants)** | | | |
| CAIDE | 1.22 (1.09, 1.38) | 1.20 (1.07, 1.36) | |
| FRS | 1.43 (1.26, 1.61) | 1.32 (1.17, 1.50) | |
| FINDRISC | 1.25 (1.10, 1.41) | 1.18 (1.05, 1.34) | |
| **Risk scores assessed at 60 years^c^ (Mean follow-up 14.0 years; 237 cases of dementia & 823 deaths in 6,944 participants)** | | | |
| CAIDE | 1.03 (0.92, 1.16) | 1.04 (0.92, 1.18) | |
| FRS | 1.23 (1.10, 1.38) | 1.16 (1.03, 1.30) | |
| FINDRISC | 1.20 (1.07, 1.36) | 1.14 (0.99, 1.30) | |
| **Risk scores assessed at 65 years^d^ (Mean follow-up 9.5 years; 210 cases of dementia & 587 deaths in 6,398 participants)** | | | |
| CAIDE | 1.05 (0.93, 1.18) | 1.07 (0.94, 1.20) | |
| FRS | 1.13 (1.01, 1.27) | 1.08 (0.96, 1.22) | |
| FINDRISC | 1.13 (1.00, 1.28) | 1.08 (0.94, 1.23) | |

CAIDE=Cardiovascular Risk Factors, Aging, and Incidence of Dementia; FRS: Framingham cardiovascular Risk Score; FINDRISC: Finnish Diabetes Risk Score.

HR: Hazard Ratio, CI: Confidence Interval

^a^**1991-1993**: 1 SD corresponds to 2.9 points in CAIDE, 3.7 points for men & 4.2 points for women in FRS, and 3.3 points for men & 3.9 points for women in FINDRISC.

**^b^Age 55**: 1 SD corresponds to 1.9 points in CAIDE, 3.4 points for men & 3.6 points for women in FRS, and 3.6 points for men & 4.0 points for women in FINDRISC.

**^c^** **Age 60**: 1 SD corresponds to 1.9 points in the CAIDE, 3.1 points for men & 3.6 points for women in FRS, and 3.8 points for men & 4.1 points for women in FINDRISC.

**^d^Age 65**: 1 SD corresponds to 1.9 points in the CAIDE, 2.9 points for men & 3.6 points for women in FRS, and 4.0 points for men & 4.4 points for women in FINDRISC.
